# Exploring Volatile Organic Compounds in Breath for High-Accuracy Prediction of Lung Cancer

**DOI:** 10.3390/cancers13061431

**Published:** 2021-03-21

**Authors:** Ping-Hsien Tsou, Zong-Lin Lin, Yu-Chiang Pan, Hui-Chen Yang, Chien-Jen Chang, Sheng-Kai Liang, Yueh-Feng Wen, Chia-Hao Chang, Lih-Yu Chang, Kai-Lun Yu, Chia-Jung Liu, Li-Ta Keng, Meng-Rui Lee, Jen-Chung Ko, Guan-Hua Huang, Yaw-Kuen Li

**Affiliations:** 1 Department of Internal Medicine, National Taiwan University Hospital, Hsin-Chu Branch, Hsin-Chu 30059, Taiwan; anskyluker@gmail.com (P.-H.T.); isan2383@yahoo.com.tw (H.-C.Y.); cjchangone@yahoo.com.tw (C.-J.C.); paukai1111@yahoo.com.tw (S.-K.L.); freeman0509@gmail.com (Y.-F.W.); ntudcm@gmail.com (C.-H.C.); pagasus2751@yahoo.com.tw (L.-Y.C.); starbox103@gmail.com (K.-L.Y.); m10082100@gmail.com (C.-J.L.); ltkeng@gmail.com (L.-T.K.); sheepman1024@gmail.com (M.-R.L.); 2 Institute of Statistics, National Yang Ming Chiao Tung University, Hsin-Chu 30010, Taiwan; superstar147896325@yahoo.com.tw; 3 Center for Emergent Functional Matter Science, National Yang Ming Chiao Tung University, Hsin-Chu 30010, Taiwan; edisonpan0805@gmail.com; 4 Department of Applied Chemistry, National Yang Ming Chiao Tung University, Hsin-Chu 30010, Taiwan

**Keywords:** volatile organic compounds, SIFT-MS, XGBoost, lung cancer, breath analysis, machine learning

## Abstract

**Simple Summary:**

Human-exhaled volatile organic compounds (VOCs) can be altered by lung cancer and become identifiable biomarkers. We used selected ion flow tube mass spectrometry (SIFT-MS) to quantitatively analyze 116 kinds of VOCs, which were exhaled by 148 lung cancer patients and 168 healthy individuals and collected from the environment to obtain a group of comprehensive data. A predictive model yielding 0.92 accuracy, 0.96 sensitivity, 0.88 specificity, and 0.98 area under the curve (AUC) was established using an advanced machine learning eXtreme Gradient Boosting (XGBoost) algorithm that considered the influences of exhaled and environmental VOCs.

**Abstract:**

(1) Background: Lung cancer is silent in its early stages and fatal in its advanced stages. The current examinations for lung cancer are usually based on imaging. Conventional chest X-rays lack accuracy, and chest computed tomography (CT) is associated with radiation exposure and cost, limiting screening effectiveness. Breathomics, a noninvasive strategy, has recently been studied extensively. Volatile organic compounds (VOCs) derived from human breath can reflect metabolic changes caused by diseases and possibly serve as biomarkers of lung cancer. (2) Methods: The selected ion flow tube mass spectrometry (SIFT-MS) technique was used to quantitatively analyze 116 VOCs in breath samples from 148 patients with histologically confirmed lung cancers and 168 healthy volunteers. We used eXtreme Gradient Boosting (XGBoost), a machine learning method, to build a model for predicting lung cancer occurrence based on quantitative VOC measurements. (3) Results: The proposed prediction model achieved better performance than other previous approaches, with an accuracy, sensitivity, specificity, and area under the curve (AUC) of 0.89, 0.82, 0.94, and 0.95, respectively. When we further adjusted the confounding effect of environmental VOCs on the relationship between participants’ exhaled VOCs and lung cancer occurrence, our model was improved to reach 0.92 accuracy, 0.96 sensitivity, 0.88 specificity, and 0.98 AUC. (4) Conclusion: A quantitative VOCs databank integrated with the application of an XGBoost classifier provides a persuasive platform for lung cancer prediction.

## 1. Introduction

Scientists have been interested in volatile organic compounds (VOCs) released by human bodies for over five decades. In 1971, Nobel Prize winner Linus Pauling revealed that breath is a complex mixture comprised of approximately 250 VOCs [1]. In 1999, Phillips et al. [2] detected over 3400 different volatile compounds in exhaled human breath. Metabolic processes of the human body produce these compounds; they enter the lungs via blood and are exhaled. Therefore, variations in exhaled breath compounds’ concentrations can be directly linked to a disease, such as cancer [3]. Of all cancer types, there are 1.6 million lung cancer deaths per year, which is more than the sum of the next three most common cancers, i.e., prostate, breast, and colon cancer [4]. Lung cancer is usually quiet in the early stages; patients frequently experience coughing, chest pain, weight loss, etc. These symptoms early tend to be ignored until advanced disease development to be taken seriously. Generally, the 5-year survival related to late diagnosis is around 10–15%. Using conventional diagnostic procedures such as computer tomography (CT), sputum cytology, and biopsy, 85% of lung cancer cases are detected at a phase at which therapy is ineffective for curing the disease [5]. Low-dose thoracic CT can detect tumors at early stages and reduce mortality from lung cancer [6]. With the incidences of lung cancer rising worldwide, early detection techniques are an essential and immediate need. Since the cost and concern regarding radiation exposure mainly impair the applications of currently available examinations, an effective, radiation-free, and less invasive approach for lung cancer screening needs to be established. Some of the current and developing methods used for lung cancer noninvasive detection methods are summarized in Table 1.

Studying VOCs is one of the most interesting strategies and has many advantages (Table 1). Researchers, commonly using gas chromatography-mass spectrometry (GC-MS), have demonstrated the presence of lung-cancer-specific profiles of VOCs [7]. Though GC-MS is an established technique for VOC analysis, compared with selected ion flow tube mass spectrometry (SIFT-MS), GC-MS requires precise calibrations of standard compounds if highly specific and reliable quantification is needed [8]. Though GC-MS analysis integrated with library search is a powerful strategy for compound identification, the quantitative assessment of VOC using GC-MS is far more complicated. On the contrary, SIFT-MS design benefits the quantitative analysis and real-time study of VOCs [9,10]. The research using SIFT-MS has been primarily centered on the esophagus and colorectal cancers [11,12], whereas the breath profile of lung cancer analysis by SIFT-MS has rarely been reported. The features of direct sampling and quantitative VOC estimation of SIFT-MS can provide a large quantity of VOC data useful for additional statistical modeling. For this kind of large data, multivariate and machine learning tools for chemometric applications seem to be the rational way to define, project, model, and interpret the results [7].

**Figure 1:**
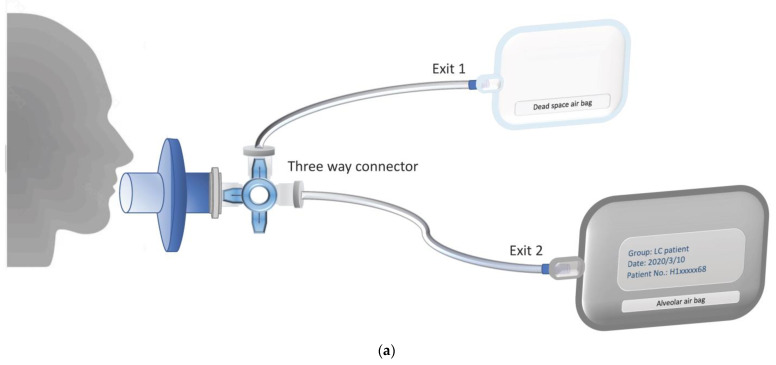

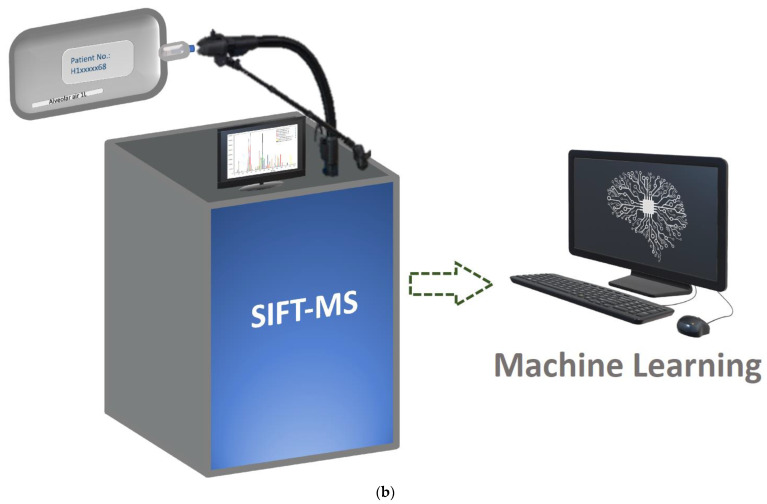
The research flow chart. (**a**) Collecting the alveolar air. The breath was exhaled through the mouthpiece with a direct-connect three-way valve. At the first stage, the exhaled air flows through Exit 1. When the volume of the front portion of the exhaled air reaches 0.2 L, the three-way valve switches to Exit 2 and starts to collect the rest of the exhaled air, i.e., alveolar air, in a 1.0 L aluminum bag. The process of sample collection may be repeated 2–3 times to collect enough samples for analysis. (**b**) Delivery and analysis of an exhaled sample. Selected ion flow tube mass spectrometry (SIFT-MS) extracts the exhaled breath from an aluminum bag and analyzes the composition of volatile organic compounds (VOCs). The VOC data are used for model construction, machine learning, and prediction of lung cancer.

The SIFT-MS technique was used in this study for the detection of VOCs in human breath from lung cancer patients and healthy volunteers. A machine learning approach named eXtreme Gradient Boosting (XGBoost) [13] was used to classify participants according to status, cancerous or healthy, based on their VOC analyses. The sampling process and flow chart of SIFT-MS analysis are shown in Figure 1.

## 2. Results

### 2.1. Characteristics of Patients with Lung Cancer and Healthy Volunteers

In this study, we enrolled 168 health volunteers (101 women) aged 20 to 74 years (controls) and 148 lung cancer patients (73 men) aged 37 to 90 years (Table 2). Lung cancer patients were older (*p* < 0.001) than the controls. Most histological types of lung cancer were adenocarcinoma (72.9%). The most elevated target driver mutation is exon 19 deletion (22.3%) and exon 21 point mutation (20.3%). Most patients had a nonresectable disease at clinical stage IIIB and C (18.2%), IVA (43.9%), or IVB (27%).

### 2.2. VOCs for SIFT-MS Analysis

**Figure 2:**
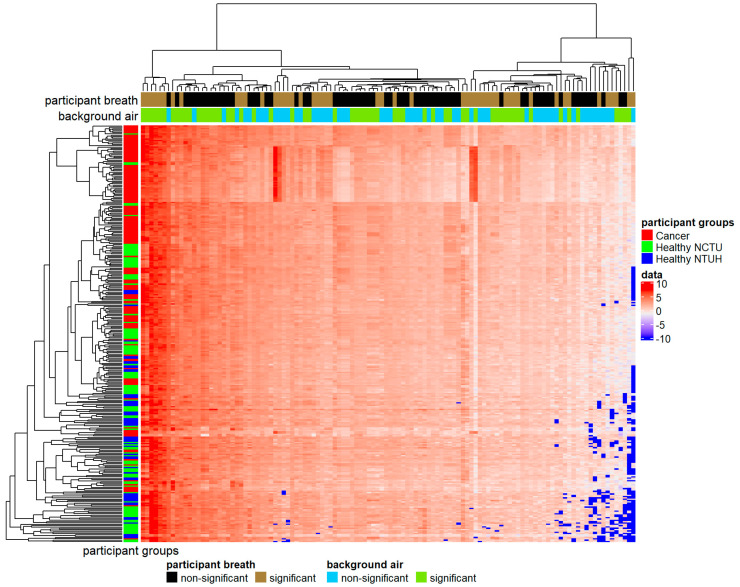
The heat map of 116 volatile organic compounds (VOC) measurements (log concentrations) for 316 participants. In the color matrix at the center, each row represents a participant, and each column represents a single VOC with the diverging color scheme of red (high concentration) and blue (low concentration). VOCs and participants are clustered using the agglomerative hierarchical clustering method. In the color bar on the left, red indicates cancer patients, green indicates healthy volunteers of the National Yang Ming Chiao Tung University (NCTU), and blue indicates healthy volunteers of the National Taiwan University Hospital Hsin-Chu Branch (NTUH). The two-color bars at the top represent the significance of VOCs. The first bar indicates whether VOCs showed significant differences between lung cancer patients and healthy controls (brown for significant and black for nonsignificant). The second bar indicates whether VOCs were significantly different between NCTU and NTUH (green for significantly different and blue for not significantly different).

This study investigated 116 specific VOCs previously reported as human breath biomarkers, shown in Table A1. Fifty VOCs showed significant differences between lung cancer patients and healthy controls in all three statistical hypothesis tests adopted (* in Table A1), which can be used as biomarkers for lung cancer. When analyzing the collected background air samples, we identified 57 environmental VOCs whose concentrations were not significantly different between the National Yang Ming Chiao Tung University (NCTU) and the National Taiwan University Hospital Hsin-Chu Branch (NTUH) in all statistical hypothesis tests (^†^ in Table A1). The heat map for VOCs of different participants is shown in Figure 2. Fifty-seven percent (*n* = 84) of cancer patients were clustered tightly together (top red in the color bar on the left). Another 24% (*n* = 36) of cancer patients and 38% (*n* = 43) of healthy volunteers of NCTU were grouped closely (middle red and green in the color bar on the left). Healthy volunteers of NTUH were more spread out (blue in the color bar on the left). The hierarchical clustering identified several VOC groups (the dendrogram on the top), where two groups contained dominant features for distinguishing between cancer cases and healthy controls. Ethanol, formic acid, ethanedial, methanol, acetone, butane, and hexane (the far-left brown in the first color bar at the top) had higher values in cancer cases than in healthy controls. Another group of VOCs, including benzoic acid and beta-caryophyllene, (the far-right brown in the first color bar on the top), showed an extremely low concentration for most healthy controls. All these dominant VOCs, except hexane, were significantly different between lung cancer cases and healthy controls. Hexane and beta-caryophyllene were not significantly different between the NCTU and NTUH.

### 2.3. XGBoost Prediction Model

For prediction modeling, we first applied XGBoost to all VOC measurements for lung cancer disease state prediction, and its accuracy, sensitivity, specificity, and area under the curve (AUC) were 0.89, 0.82, 0.94, and 0.95, respectively. Using XGBoost with 50 significantly different VOCs between lung cancer cases and healthy controls, we obtained accuracy, sensitivity, specificity, and AUC of 0.90, 0.84, 0.94, and 0.94, respectively, demonstrating the efficacy of our list of potential lung cancer biomarkers. Notably, our sensitivity and specificity were different, indicating the model’s differential prediction ability for lung cancer cases and healthy controls. This might be due to the confounding effect from environmental VOCs, where all cases’ breath samples were taken in the hospital, whereas those from controls were taken either in the hospital or in the academic campus.

### 2.4. Adjust Algorithm for Environmental VOCs

When XGBoost was built on environmentally nondifferential VOCs to eliminate the potential confounding effect from environmental VOCs, the accuracy, sensitivity, specificity, and AUC were 0.88, 0.84, 0.90, and 0.92, respectively, representing a slight improvement in sensitivity but a deterioration in specificity. We further applied SMOTE [33] to the VOC values of the collected background air samples to create synthetic environmental VOCs for each participant. The XGBoost prediction model incorporating participants’ exhaled VOCs and these simulated environmental VOCs can account for nonendogenous VOCs present in the environment. This model achieved better performance with 0.92 accuracy, 0.96 sensitivity, 0.88 specificity, and 0.98 AUC. These results are consistent with our speculation concerning the confounding effect of environmental VOCs. The approaches adopted here which consider environmental VOC effects can also improve prediction accuracy.

## 3. Discussion

Each whole breath can be divided into three parts according to the pressure of CO_2_ in the exhalation. The first and second parts are dead space from the oropharynx and upper respiratory tract. The third part is the air from the alveoli deep inside the lungs that can exchange gases with the blood [34]. Previous breath analyses of lung cancer were performed by collecting the whole breath [35,36]. Some studies collected end-tidal breath by discarding the front of the breath [37,38] or filling the dead space air into other bags [39]. Studies have shown that the concentration of VOC is different in whole breath and end-tidal breath [40]. Our study used three-way connectors to manually fill Tedlar bags with air from dead areas of the mouth and upper respiratory tract and subsequently collect alveolar air from deep in the lungs into aluminum bags. Since VOCs in the alveoli are derived from the blood and gas exchange within the alveoli, this approach can better reflect VOCs’ relationship with metabolic state changes caused by disease physiology.

The influence of environmental VOCs at the time of sampling can be considerable in the breathomics of lung cancer. More than 1000 exogenous VOCs are known to be detected in human respiration [41]. The relationship between environmental VOCs and the human body is complex and involves the processes of mixing, diffusion, and distribution in the blood and the metabolism in adipose tissue [42]. The concentration, exposure time, and solubility of the environmental VOCs in the human body and the individual physiology are the important factors that significantly affect the VOC contents of exhaled breath [43]. In past studies, there were no generally applicable rules for considering the influence of environmental VOCs. In addition to using the alveolar gradient concept [44], researchers solved this problem using inspiration filters [45] or having patients spend some time in the ventilation room before collection [38]. Although these methods are effective and widely accepted, we take one step further to eliminate the possible variances caused by environmental factors. Herein, we successfully introduced new algorithms to simulate environmental VOCs at the sampling time and incorporated them into the model to improve prediction accuracy. We showed that our approach could further abrogate the perturbation from environmental VOCs. For further applicability in various environments in the future, we suggest collecting and analyzing VOCs from participants and the environment simultaneously. The XGBoost model can further proceed with the process of learning and tuning, thus minimizing confounding effects. We significantly improved lung cancer prediction accuracy by selecting the phase of breath and calibrating environmental VOCs’ impact.

This study describes an innovative machine-learning-based approach that uses SIFT-MS quantitative data to accurately distinguish respiratory samples of lung cancer patients from healthy controls. SIFT-MS quantitative analysis is achieved by applying precisely controlled ultra-soft chemical ionization combined with mass spectrometry detection [8]. The advantage of direct SIFT-MS is that it simplifies the needs for sample preparation, preconcentration, and chromatography. Our study shows that this machine-learning-based breath test is 0.96 sensitive, 0.88 specific, and highly accurate (0.98 area under the curve (AUC)) for identifying lung cancer, whereas the previous studies using multivariate classifiers based on VOCs’ chemometrics analyzed by GC-MS demonstrated moderate to high accuracy (AUCs of 0.63–0.9, Table 3). The models reported herein show several advantages. The quantitative characteristics of SIFT-MS are attractive because many quantitative data can enhance model development and fine-tune the XGBoost model to improve prediction accuracy. The adopted XGBoost has been well-recognized and successfully applied in big data analytics. More importantly, our strategy of incorporating environmental VOC factors has unequivocally enhanced the power of XGBoost modeling for prediction.

Limitations of this study include the fact that it was a single-center and case–control study, and most of the patients were elderly and with advanced lung cancer. Our lung cancer patients were significantly older than healthy controls, leading to age mismatches and bias in case–control studies. With aging, a higher degree of oxidative stress occurs, and levels of VOCs in the breath increase, such as isoprene, alkanes, and methylated alkanes [46,47]. Our prediction model built on older and late-stage patients may fail in early detection of the disease. A multicenter study is currently planned to collect young, early, and operable lung cancer patients, aiming to provide an effective approach for early detection of lung cancer and a definitive answer to the test’s accuracy.

The clinical application of breathomics in lung cancer remains challenging up to the present. There are considerable differences in respiration sampling procedures, study designs, and data analysis methods implemented by studies for breathomics of lung cancer, which lead to inconsistent results. The effect of nutritional habits on breath VOCs can be complicated. By modifying metabolism, inflammation, or redox status, or communicating with gut flora, food influences breath VOCs. However, how long it takes for the dietary VOCs to be removed from the breath is not known. The dietary style also has a sustained influence that fasting could not remove [31]. There is no consensus on how to eliminate these dietary effects. We thus did not strictly screen participants’ nutritional status because we wanted to collect data of different dietary habits to establish a big data model that can be universally applied to the general population in the future. Another issue is that there is no validated list of VOC lung cancer biomarkers in the literature [31,48]. The lung cancer biomarkers found in these studies are mostly inconsonant [49]. The mechanism of most VOCs exhaled by the human body remains unclear. The following factors affect the concentration and composition of lung cancer VOCs in the human body: oxidative stress, cytochrome P450, liver enzymes, metabolic carbohydrates (glycolysis/gluconeogenesis pathways), and lipid metabolism [32]. These possible biochemical pathways vary from person to person, resulting in increased or decreased volatile organic compounds concentrations. Under these complex mechanisms, biochemical pathways of VOCs research cannot provide a definite answer. These VOCs that are considered possible and focused can be classified into the following families: hydrocarbons, primary and secondary alcohols, aldehydes and branched aldehydes, ketones, esters, nitriles, and aromatic compounds [32]. Our list of the targeted 116 VOCs was mainly derived from a literature search [32,49]. In this paper, we detail our approaches for VOC breathomics and provide suggested guidelines for further studies. Based on VOC analysis, this is by far the most comprehensive study (Table 3). Our research outcome provides a potentially useful model and platform for nonradiative and noninvasive lung cancer diagnosis.

## 4. Materials and Methods

### 4.1. Study Participants and Data Collection

Between May 2019 and June 2020, we obtained breath samples from 148 patients with histologically confirmed lung cancers and 168 healthy volunteer staff. All breath samples of lung cancer patients were collected in the National Taiwan University Hospital Hsin-Chu Branch (NTUH), and 112 and 56 healthy volunteers had their breath samples collected in the National Yang Ming Chiao Tung University (NCTU) and NTUH, respectively. Although we collected multiple breath samples from each healthy volunteer of the NCTU, only one randomly selected sample of each volunteer was included in the flowing analyses. The healthy volunteers had no history of significant pulmonary disease in any other organ and were free of disease. The patients’ demographic profiles, history of cigarette smoking, staging, pathological finding, and cancer mutation testing were retrospectively collected from their medical records. The clinical cancer stage was based upon the American Joint Committee on Cancer (AJCC) TNM staging system 8th edition [54]. To evaluate the confounding effect from environmental VOCs, we also collected 18 and 29 background air samples from the NCTU and NTUH, respectively. These environmental VOCs were collected in the same place on the same day as participants’ VOCs were obtained. All collected VOCs of the environment and participants were then analyzed. Each patient and volunteer provided written informed consent. This research was approved by the National Taiwan University Hospital Hsin-Chu Branch institutional review board (108-023-E).

### 4.2. Breath Sampling Methodology

All participants orally rinsed with water before sampling for breathing and stayed in the same place for more than 30 min before collecting the gas. To collect the alveolar breath and remove dead space air, each participant breathed normally through a disposable mouthpiece and into the device. We collected 0.2 L of the front portion of the exhaled breath flow through Exit 1 into a Tedlar bag (SKC Inc., Eighty Four, PA, USA), controlled by a 3-way valve. The remaining part of the exhaled breath, alveolar air, was collected in a 1.0 L aluminum bag, as shown in Figure 1a. A bag of room air was collected concurrently with some breath samples to account for nonendogenous VOCs present in the environment. Before collection, aluminum breath analysis bags were flushed with nitrogen gas at least ten times to remove background VOCs associated with the bags. The sealed samples were kept at room temperature (25 °C) and analyzed within 6 h. (Figure 1b). In some reports [55,56,57], VOC storage in bags potentially causes VOC content changes in breath samples due to storage and transportation conditions. In our case, we collected the samples from the location within a 15-min driving distance. To confirm our storage condition’s feasibility, we performed a time-dependent analysis (twice a day for three days) on ten breath samples. By comparing the quantitative data from each sample, we conclude that most VOCs are considered stable.

### 4.3. Measurements of VOCs in Exhaled Air

The SIFT-MS theory is based on direct mass spectrometric analysis of VOCs in air or vapor samples by chemical ionization. Selected precursor ions (H_3_O^+^, NO^+^, and O_2_^+^) are injected into the helium carrier gas and ionize the VOCs in the breath samples, generating characteristic productions detected by the downstream quadrupole mass spectrometer. Real-time quantification is achieved by measuring the count rate of both precursor ions and the characteristic product ions in the downstream detection system. The concentration of trace and volatile compounds is achieved at the parts-per-billion or parts-per-million by volume. It enables the simultaneous quantification within a gaseous mixture of several VOCs. A SIFT-MS instrument (VOICE200 ultra, Syft Technologies, Christchurch, New Zealand) was used to analyze the exhaled breath samples applying the selective ion mode (SIM). The 116 compounds shown in Table A1 were separated into seven categories: alkanes, ketones, aldehydes, alcohols, amines, thiols, and others for quantitative analysis. Among these VOCs, some of the product ions from different VOCs, e.g., MH^+^ ions at No. 43, 47, 57, 59, 60, 61, 69, 71, 75, 85, 87, 89, 91, 97, 99, 101, and 103, overlap with others. The quantitative estimations of the selected VOCs were performed based on the pre-set protocol of SIFT-MS. This setting combines several product ions derived from three different reagent ions, H_3_O^+^, O_2_^+^, and NO^+^, with a tolerance feature setting at 20%. The tolerance feature is employed to deal with product ion interferences for every single compound. The final analyte concentration is calculated as an average of the lowest product ion concentration and anything within 20%. Any product ion that falls beyond the 20% tolerance range will not be accounted for in the calculation. Note that part of VOCs with the interference of product ions cannot be resolved by tolerance feature set can only be quantified as a relative scale. Both the accurate measurements and the VOCs’ relative scales are collected and employed to construct statistical models.

### 4.4. Statistical Analysis

All analyses were performed using the R software (version 4.0.2; R Foundation for Statistical Computing). Participants’ characteristics were analyzed and compared between lung cancer patients and healthy controls using the t-test or Wilcoxon rank-sum test for continuous variables and the Chi-square test or Fisher’s exact test for categorical variables.

The heat map [58] was used to visualize VOCs’ changes in different participants, helping establish the initial research hypothesis. We used the Wilcox rank-sum test and two-sample t-test with or without equal variance assumption to determine whether VOCs’ differences between lung cancer patients and healthy controls were significant. To correct for multiple comparisons, the significance of the difference for each of the 116 VOC measurements was assessed at Bonferroni-corrected *p*-value = 0.0004 (0.05/116) [59]. We used the XGBoost [13], a machine learning method, to build a prediction model that used VOC measurements to predict lung cancer’s disease state. We used 70% of the collected VOC data as the training set to build the prediction model and then used the other 30% to test its performance. The prediction model was established based on either all VOC measurements or those with a significant difference between the two study groups. To eliminate the confounding effect from environmental VOCs, we tried out two approaches: the prediction model built on VOCs whose concentrations were not significantly different among different environments and the model incorporating participants’ exhaled VOCs and corresponding environmental VOCs simulated via SMOTE [33] from collected background air samples. The performance of various approaches was evaluated in terms of accuracy (the proportion of correctly classified participants), sensitivity, specificity, and AUC (the area under the ROC curve) on the test data.

## 5. Conclusions

To summarize, the machine learning model proposed in this study can accurately identify lung cancer using participants’ exhaled breath. It is a non-invasive and radiation-free system, which can accelerate the diagnosis of lung cancer. We successfully demonstrated a new approach for disease diagnosis by integrating several techniques, including comprehensive and quantitative VOC analysis and deep learning algorithms for minimizing the interference of environmental factors, which resulted in an accurate prediction model. The development of standardized and automatic breath sampling protocols is an ongoing project that we expect will vastly simplify the process of sample collection and guarantee sample quality. We are confident that these efforts will ultimately unlock the potential and importance of human breathomics.

## Figures and Tables

**Table 1:** Summary of the noninvasive detection methods for lung cancer.

Biomarkers/Specimen	Analytic Platform	Detection Target	Sensitivity (%)	Advantages	Deficiencies	Ref.
CTCs/Blood	IF; FISH	EpCAM, Size-based cells	30.0–69.5	Viable cell, high specificity, high throughput	Limited sensitivity; require enrichment; only detect advanced cancers	[14,15]
Traditional Proteins/Blood	ECLIA	CEA, CYFRA 21-1	22–69	Rapid and common	Limited sensitivity and specificity	[16]
Novel Proteins/EBC, Saliva, Urine, Blood	Microarray; LC-MS/MS	CKAP4, exosomal proteins (NFX1, PKG1, GPC1)	70.0–84.0	Higher sensitivity; high throughput; rapid	Quantity required (MS); validation required	[17,18,19]
microRNA/Blood	Microarray; RT-PCR; NGS	miRNAs-126, -145, -210 and -205-5p, -17, -190b, -19a, -19b, -26b, -375	80.0–91.5	High throughput, stable	Specialized abilities and facilities are required	[20,21,22,23,24]
Methylated DNA/Blood	NGS; PCR	HOXD10, PAX9, PTPRN2, STAG3, SHOX2	70.0–87.8	High sensitivity and specificity	Require standardization	[25,26,27]
ctDNA/Blood	NGS; Multiplex-PCR	Genetic mutation, SNVs	48.0–59.0	Target for precision medicine; early detection (~70 days prior to CT image)	Limited sensitivity, require expensive equipment	[28,29,30]
VOCs/Exhaled Breath	E-Nose sensors; GC-MS; PTR-MS, IMS; LPPI-MS	propanol, isoprene, acetone, pentane, hexanal, toluene, benzene, ethylbenzene, and others	81.0–96.5	Rapid, simple, noninvasive; inexpensive	Require standardization	[7,31,32]

Abbreviations: CTCs (circulating tumor cells); IF (immunofluorescence); FISH (fluorescence in situ hybridization); EpCAM (epithelial cell adhesion molecule); ECLIA (electrochemiluminescence immunoassay); CEA (carcinoembryonic antigen); CYFRA 21-1 (cytokeratin fraction 21-1); EBC (exhaled breath condensate); NGS (next-generation sequencing); CT(computed tomography); RT-PCR (reverse transcription PCR); ctDNA (circulating tumor DNA); SNVs (single-nucleotide variants); GC-MS (gas chromatography mass spectrometry); PTR-MS (proton transfer reaction mass spectrometry); IMS (ion mobility spectrometry); LPPI-MS (low-pressure photoionization mass spectrometry); VOCs (volatile organic compounds).

**Table 2:** Characteristics of patients with lung cancer and healthy volunteers of the study.

Characteristic	Lung Cancer Patients (*n* = 148)	Health Controls (*n* = 168)
Age (years), y *		
Mean ± SD	64.5 ± 11	31.4 ± 10.4
Rage	37–90	20–74
Sex, *n* (%) ^†^		
Female	75 (50.7)	101 (60.1)
Male	73 (49.3)	67 (39.9)
Smoking status, *n* (%) *		
Current smoker	9 (6)	0
Former smoker	47 (31.2)	1
Nonsmoker	92 (62.1)	167 (99)
Lung cancer type, *n* (%)		-
Adenocarcinoma	108 (72.9)	
Squamous cell carcinoma	17 (11.5)	
Small cell lung cancer	14 (9.5)	
Other lung cancer	8 (5.4)	
Targetable driver mutation, *n* (%)		
EGFR		-
Exon 19 deletion	33 (22.3)	
Exon 21 point mutation	30 (20.3)	
T790M	6 (4.1)	
ALK	7 (4.7)	
ROS1	3 (2.0)	
Wild type	75 (50.7)	
PD-L1 expression, *n* (%)		
>50%	18 (12.1)	-
1–49%	57 (39.0)	
<1%	29 (19.6)	
Clinical stage status, *n* (%)		
IA and B	4 (2.7)	-
IIA and B	4 (2.7)	
IIIA	8 (5.4)	
III B and C	27 (18.2)	
IVA	65 (43.9)	
IVB	40 (27.0)	

* Significantly different between lung cancer patients and healthy controls at *p*-value < 0.05. ^†^ Significantly different between lung cancer patients and healthy controls at *p*-value < 0.1. Abbreviations: EGFR (epidermal growth factor receptor); ALK (anaplastic lymphoma kinase); ROS1 (ROS1 oncogene); NTUH (National Taiwan University Hospital Hsin-Chu Branch); NCTU (National Yang Ming Chiao Tung University).

**Table 3:** Summary of various algorithms applied to lung cancer diagnosis using multiple VOCs.

Algorithms	Analytical Platform	Patients with Cancer No.	Analyzed VOC No.	Sensitivity %	Specificity %	AUC	Reference/(Year)
Stepwise Discriminant Analysis	GC-MS	67	9	85.1	80.5	NR	[35]/(2003)
Logistic Regression	GC-MS	193	16	84.6	80.0	0.88	[50]/(2007)
Weighted Digital Sum Discriminator	GC-MS	193	30	84.5	81	0.9	[32]/(2008)
Support Vector Machine	GS-MS	107	5	95	89	NR *	[51]/(2016)
Artificial Neural Networks	GC-MS	108	88	86.36	86.36	0.86	[52]/(2019)
K-nearest Neighbor	GC-MS	325	NR	NR	NR	0.63 ^†^	[53]/(2020)
Extreme Gradient Boosting	SIFT-MS	148	116	82	94	0.95	This WorkConsidering only participants’ VOCs
				96	88	0.98	Considering both participants’ VOCs and environmental VOCs

Abbreviations: AUC, area under the curve; GC-MS, gas chromatography-mass spectrometry; NR, not reported; SIFT-MS, selected ion flow tube mass spectrometry; * Accuracy: 89%, ^†^ Classify adenocarcinoma and squamous cell carcinoma patients.

## Data Availability

The data presented in this study are available on request from the corresponding author. The data are not publicly available due to technical limitations, the potential issue of intellectual property, and ethics.

## Appendix A

**Table A1:** The 116 VOCs selected for selected ion flow tube mass spectrometry analysis of breath samples in this study.

No.	Compound	No.	Compound	No.	Compound	No.	Compound
1 *^,†^	beta-caryophyllene (87-44-5)	30	2-pentanone (107-87-9)	59	diethyl ether (60-29-7)	88 ^†^	1,4-diaminobutane (110-60-1)
2	pyrrole (109-97-7)	31 *^,†^	(E)-2-heptenal (18829-55-5)	60	isobutyl alcohol (78-83-1)	89	o-xylene (95-47-6)
3 *	benzoic acid (65-85-0)	32 ^†^	3-buten-2-one (78-94-4)	61 ^†^	2-methylpentane (107-83-5)	90 ^†^	cyclopentane (287-92-3)
4 *^,†^	2,5-dimethylfuran (625-86-5)	33 ^†^	butanone (78-93-3)	62	methylcyclopentane (96-37-7)	91	propane (74-98-6)
5 *	acetophenone (98-86-2)	34 *^,†^	1,5-diaminopentane (462-94-2)	63 ^†^	heptanal (111-71-7)	92	heptane (142-82-5)
6	pyridine (110-86-1)	35 *^,†^	alpha-terpinene (99-86-5)	64	1-butanol (71-36-3)	93	propanal (123-38-6)
7 *	2-methylpyrazine (109-08-0)	36 *	1-butyne (107-00-6)	65	3-methyl-2-butenal (107-86-8)	94 *	2-propanol (67-63-0)
8 ^†^	tridecane (629-50-5)	37	1-methyl-2-pyrrolidinone (872-50-4)	66 ^†^	pentanoic acid (109-52-4)	95 *^,†^	cyclohexane (110-82-7)
9 ^†^	2,5-dimethylpyrazine (123-32-0)	38 ^†^	diisopropyl ether (108-20-3)	67 *	ethylbenzene (100-41-4)	96	ethane (74-84-0)
10 ^†^	1,3-butadiene (106-99-0)	39	2-pentanone new (107-87-9)	68 *^,†^	1-heptene (592-76-7)	97	carbon disulfide (75-15-0)
11 *^,†^	dodecane (112-40-3)	40 *	1,2,4-trimethylbenzene (95-63-6)	69 *^,†^	dimethyl sulfide (75-18-3)	98 *^,†^	trimethylamine (75-50-3)
12	propyne (74-99-7)	41 ^†^	nonane (111-84-2)	70 *^,†^	propanoic acid (79-09-4)	99	acetaldehyde (75-07-0)
13 ^†^	(E)-2-nonenal (18829-56-6)	42 *	propylbenzene (103-65-1)	71	toluene (108-88-3)	100	dimethyl ether (115-10-6)
14 *	4-isopropyl toluene (99-87-6)	43	3-butyn-2-ol new (2028-63-9)	72	p-xylene (106-42-3)	101 ^†^	acetic acid (64-19-7)
15 ^†^	2-hexanone (591-78-6)	44 *^,†^	cyclohexanone (108-94-1)	73 ^†^	3-methylbutanal (590-86-3)	102	propene (115-07-1)
16	undecane (1120-21-4)	45 *^,†^	ethylcyclohexane (1678-91-7)	74	butanal (123-72-8)	103	formaldehyde (50-00-0)
17 *	benzaldehyde (100-52-7)	46 ^†^	2-methylbutanal (96-17-3)	75	xylenes + ethylbenzene (1330-20-7)	104	furan (110-00-9)
18 *	styrene (100-42-5)	47 *	nonanal (124-19-6)	76 *	isopropylamine (75-31-0)	105 *	1-propanol (71-23-8)
19 *^,†^	eucalyptol (470-82-6)	48 *^,†^	limonene (138-86-3; 7705-14-8)	77 *^,†^	methyl acetate (79-20-9)	106 ^†^	isobutane (75-28-5)
20 ^†^	furfural (98-01-1)	49 ^†^	2-pentene (109-68-2)	78 *^,†^	1-hexene (592-41-6)	107	isoprene (78-79-5)
21 *	1-pentanol (71-41-0)	50	decane (124-18-5)	79 *^,†^	1-butene (106-98-9)	108 *	formic acid (64-18-6)
22 *^,†^	butyl acetate (123-86-4)	51	methyl n-propyl sulfide (3877-15-4)	80 ^†^	pentanal (110-62-3)	109	pentane (109-66-0)
23 *	octanal (124-13-0)	53 ^†^	2-methylpropanal (78-84-2)	81	1-methoxy-2-propanol (107-98-2)	110 *	acetonitrile (75-05-8)
24 *	3-methyl-1-butanol (123-51-3)	53 *^,†^	acetoin (513-86-0)	82	2,3-butanediol (513-85-9; 513-89-3)	111 *	ethanol (64-17-5)
25 ^†^	(E)-2-hexenal (6728-26-3)	54 *^,†^	alpha-pinene (80-56-8; 2437-95-8)	83 ^†^	hexanal (66-25-1)	112 ^†^	hexane (110-54-3)
26 ^†^	1,4-butyrolactone (96-48-0)	55 *	acrylonitrile (107-13-1)	84 *^,†^	acrolein (107-02-8)	113 *	methanol (67-56-1)
27 ^†^	6-methyl-5-hepten-2-one (110-93-0)	56 *^,†^	ethyl acetate (141-78-6)	85 ^†^	acetic anhydride (108-24-7)	114 *	acetone (67-64-1)
28	benzene (71-43-2)	57 *^,†^	2,3-butanedione (431-03-8)	86 ^†^	3-methylpentane (96-14-0)	115 *	butane (106-97-8)
29 ^†^	decanal (112-31-2)	58 *^,†^	2-methyl-2-propenal (78-85-3)	87 *^,†^	octane (111-65-9)	116 *	ethanedial (107-22-2)

* The VOC that showed a significant difference between lung cancer patients and healthy controls in all three statistical hypothesis tests adopted. ^†^ VOCs whose concentration was not significantly different between the National Yang Ming Chiao Tung University (NCTU) and the National Taiwan University Hospital Hsin-Chu Branch (NTUH) in all statistical hypothesis tests adopted.
